# A comparative analysis of the chemical composition and compliance level to established standards of concrete reinforcement steel rods rolled in Nigeria

**DOI:** 10.1016/j.heliyon.2022.e09597

**Published:** 2022-05-30

**Authors:** Richard O. Leramo, Lawson O. Adekoya, Oluwaseun Kilanko, Sunday O. Oyedepo, Stephen E. Eluwa, Phillip O. Babalola, Samson O. Adeosun, Nicholas T. Leramo, Peter O. Aiyedun, Olayinka S. Ohunakin, Oluseyi O. Ajayi, Ojo S.I. Fayomi

**Affiliations:** a Mechanical Engineering Department, Covenant University, Ogun State, Nigeria; b Mechanical Engineering Department, Obafemi Awolowo University, Ile-Ife, Osun State, Nigeria; c Orient Concept, Jamade Quarters, Apete Ibadan, Oyo State, Nigeria; d Metallurgical and Materials Engineering Department, University of Lagos, Nigeria; e Nigeria Security and Civil Defence Corps, Oyo State Command, Ibadan, Nigeria; f Mechanical Engineering Department, University of Agriculture, Abeokuta, Nigeria

**Keywords:** Chemical composition, Steel reinforcing bars, Locally-rolled, Billets, Scraps, Standards

## Abstract

Nigeria is presently facing the challenges of collapsing buildings and bridges due to substandard materials used as reinforcement products. The increasing use of scraps as feedstock for the production of reinforcing steel bars by steel rolling mill companies has adversely affected the quality of rebars in Nigeria. This research study aimed to appraise the chemical properties of selected brands of steel rebars of Nigeria. Thirty selected brands of rebars were sourced from the six geopolitical zones in Nigeria, and their chemical compositions were analysed for level of compliance with five selected standards (SON, BSI, ASTM, AISI, ISO). The chemical composition test was performed using Optical Light Spectrometric methods. One way analysis of variance (ANOVA) test was performed using SPSS version 20 to examine whether significant differences exist or not in mean chemical composition for the different categories of selected steel rods. Statistical analysis shows a significant difference (P < 0.05) in chemical composition and compliance level between the different types of selected steel rods. The imported steel rods recorded the highest mean (μ = 101.4) in terms of chemical composition and compliance, followed by locally rolled from imported billets (μ = 101.2), TMT steel rods (μ = 101.0), and ordinary steel rods (μ = 100.6). Concerning CEV_1_ and CEV_2_, it was observed that all the brands were fully compliant within the maximum permissible ranges given in the local, foreign and international standards except an ordinary steel bar of Brand 16, which has value beyond the specified limits of CEV_1_. This study also shows that all imported and 77.8% of locally-rolled steel bars are low-carbon steel as specified by the selected standards.

## Highlights

• Findings from this study show that at a 95% confidence level, there is a significant difference (P < 0.05) in chemical composition and compliance level between the different categories of selected steel rods. The imported steel rods recorded the highest mean (μ = 101.4) in terms of chemical composition and compliance, followed by locally rolled from imported billets (μ = 101.2), TMT steel rods (μ = 101.0), and ordinary steel rods (μ = 100.6).
• The study discovered discrepancies in the values of elements (chemical compositions) in the steel brands available in Nigerian markets due to the non-compliance of some rebars' brands with standards specifications for steel bars for concrete reinforcement.
• Results revealed that the imported steel bars met most of the standard specifications, followed by locally-rolled steel bars produced from imported billets after this was, the TMT steel bars. In contrast, the ordinary steel bars produced from billets made from local scraps did not meet most standards specifications.
• All brands were fully compliant within the maximum permissible CEV ranges given in the local and international standards except Brand 16 (an ordinary steel bar), which has value beyond specified limits.
• The results showed that all imported and approximately 77.8% of locally-rolled steel bars were low-carbon steel which complied with specifications by all the standards.
• Only some chemical elements of steel have specifications in the standards. Whereas, according to the chemistry of steel rods, those unmentioned elements may have an immense contribution to the service life of the rods even if they were only present in minute quantities.

## 1 Introduction

A reinforcing concrete structure is a composite of reinforcing bars and concrete. Concrete comprises Portland cement, aggregate (rock, sand, or gravel), and water. The reinforced concrete structure is mainly aimed to withstand induced forces [1]. Concrete alone is known to be excellent in compression but very weak in tension. To compensate for this concrete shortcoming, the introduction of steel reinforcing rods that have better strength to withstand tensile forces is usually employed. The quality of a steel reinforcing rod can largely be determined by its chemical elements’ constituent. Steel reinforcing bars (rebars) for construction and building purposes in Nigeria are acquired from local and imported sources. The imported steel bars are mainly from Russia, Ukraine and Turkey [2]. The local rebars are produced from locally sourced scraps because it is economically advantageous compared to importing them into the country. This is because the two integrated iron and steel plants (Ajaokuta and Delta Steel companies) that produce billets to feed inland rolling mills are not functional [2, 3]. The growing use of scraps (motor parts, outmoded machinery, cans, roofing sheets, moulds, etc.) as feedstock for the production of reinforcing bars by steel rolling mills in Nigeria has poorly affected the quality of obtainable rebars in the Nigerian markets [3, 4]. Therefore, the chemical and mechanical properties of scrap-recycled steel rods cannot be ascertained due to non-conformity with local, foreign and international standards. This means the design of reinforcement steel bars in concrete may not be reliable for all types of buildings and constructions [5, 6].

Many researchers have discussed the use of locally produced iron bars in the construction industry in Nigeria. Ede et al. [7] and Adigun et al. [8] explained that most construction companies in Nigeria purchase their rebars from open local markets that cannot offer technical information concerning their products and their conformity with the elemental requirement from standards. Therefore, the integrity of these available steel bars in the local markets has to be ascertained to avoid unwanted circumstances that might emanate from using substandard rebars. The prime reason why the chemical composition of steel bar for reinforcement of concrete have to conform with standards is that, it has significant effect on the strength, ductility, weldability, cold or hot-shortness, mechanical and microstructural properties [9]. The only principal elements considered by the local, foreign and international standards for the maximum permissible value of rebars are Carbon (C), Phosphorus (P), Sulphur (S), Copper (Cu), Magnesium (Mn), Silicon (Si), Chromium (Cr), Molybdenum (Mo), Vanadium (V) and Nickel (Ni). Some of these elements are used to determine steel rebars' Carbon Equivalent Values (CEV). The presence of Carbon can increase strength, hardness, wear resistance, austenitic stability, and decrease ductility. Likewise, Phosphorus can cause cold-shortness while Sulphur, Copper, and Zinc can cause hot shortness if not adequately controlled [10]. These advantages and disadvantages of elemental chemical composition present in different proportions in reinforcement rods have to be appropriately regulated via standard regulations for structural integrity and the eventual safety of lives and properties.

Many researchers have contributed immensely to studying the composition of chemical elements present in reinforced steels for concrete in Nigeria*.* Odusote and Adeleke [11] stated that none of the reinforcement steel bars obtained from three collapsed building sites in Lagos, Nigeria conformed to the maximum limits recommended by BS 4449 Grade 460B and ASTM A706 standards in terms of their carbon, sulfur, and phosphorus contents. Their study also revealed that brittle globules of FeS and Fe_3_P were present in the specimens' microstructure. This brittleness was due to higher contents of harmful Sulphur and Phosphorus. In their study, Adeleke et al. [12] compared the elemental composition of the selected samples of steel rods with BS 4449, reported that 90.9%, 36.4%, and 72.7% of the samples did not conform to the maximum limits of Carbon, Sulfur, and Phosphorus respectively. Besides, only Copper was 100% compliant out of the tested samples. These trends were also supported by Balogun et al. [13], Adeleke and Odusote [14], and Alabi and Onyeji [15]. Ponle et al. [16] studied the chemical constituent of six locally manufactured steel bars from scraps and two imported steel bars with their results were compared with the specification of Grade 420 for unwelded reinforcement bars document in NIS 117:2004. The results showed that all the tested samples from locally produced bars complied with the expected standard for Manganese and Aluminum. None conformed with Sulphur and Phosphorus specifications. Only 83.3% of locally produced bars were within specifications of Carbon and Silicon. The imported bars complied with specifications for Silicon, Manganese, and Aluminum, none were within ranges for Sulphur and Phosphorus. The imported bar sample was below the maximum value for Copper. Akinsola et al. [17] characterized the low carbon steel bar used for reinforcing animal house buildings. The study showed that the percentage of Carbon and other elements such as Phosphorous, Sulphur, Manganese, Silicon, Copper, and Nitrogen agreed with the NIS 117:1992 standard. Ocheri and Ibe [18] conducted a comparative assessment of locally produced reinforcing steel bars for structural purposes. Their study disclosed that none of the three selected samples has carbon content up to the maximum permissible values in BS 4449:1997 and ASTM A706 standards documents. The study revealed that other alloying elements, such as Mn, Si, Cu, S, P, Cr, were within the standard specifications.

In the study carried out by Adigun et al. [8] on the assessment of the properties of reinforcing steel bars used in the construction industry within Lagos and its environs, they found that the elemental composition of the specimen tested in 2014 had the Carbon percentages ranged from 0.108 to 0.205 and 0.120 to 0.200 for local and imported rebars respectively. The same trend of results was shown in 2015 examinations, where the Carbon percentage composition varied from 0.110 to 0.207 and 0.110 to 0.210 for local and imported rebars, respectively. The Sulfur and Phosphorus contents of both local and imported samples were within the permissible values stated by NIS 117-1992 and BS 4449:1997 standards. Shuaib-Babata et al. [19] evaluated reinforcement steel bars of 16 different samples commercially available in sizes of 10, 12, and 16mm. Results of the study were compared with NIS 117:2004, BS 4449:2005, ASTM A615-73, and AISI 1018 standards. Their elemental compositions results uncovered the inconsistencies and discrepancies in the qualities of the reinforcement rods available in Nigeria. Conversely, the quality of reinforcement rods obtainable from another location in Nigeria was evaluated. The results showed that some products did not conform with NIS 117:2004 standard [20]. It was reported that there was excess existence of impurities, as evidenced by the percentage compositions of Sulphur, Phosphorus, and Silicon in most of the tested samples rebars in Nigeria [21]. Ajagbe et al. [22] reported that some of the reinforcing steel bars available in Nigeria do not conform with the requirements of the percentage chemical composition specified in the local (NIS 117:2004) and international standards (ISO 6935-2:2007).

Awofadeju et al. [23] assessed the chemical composition of four (4) different reinforced steel bars available in Nigerian used for structural purposes. One was imported steel bars among the tested samples, while three were rolled locally. A chemical composition assessment was carried out on 12 and 16 mm of each brand. The study showed that only 16mm imported steel bars conformed with the stipulated maximum permissible value for carbon in BS4449, ASTM A706, and NST65-Mn standards, while 50% of locally produced steel bars conformed with BS4449 and ASTM A706. About 83.3% of local steel bars complied with NST65-Mn. The study further revealed that none of the tested specimens falls within the permissible value for Sulphur. Also, the 16mm sample of the imported bars and one of the 16mm locally produced bars were within the specified value for Phosphorus in the BS4449 standard. The 12mm imported steel bars and other locally produced steel bars did not comply with ASTM A706 and NST65-Mn standards. Odusote et al. [24] affirmed that by comparing the chemical composition of the samples from different rolling mills plants with various local (NIS 117) and foreign (BS4449 and ASTM A706) standards, there were inconsistencies in Carbon, Sulphur, Phosphorus, and other elements content. Anyanwu et al. [25] carried out a study on the compliance of the mechanical properties of the reinforcement steel rods available in Imo state markets with the BS8110 design standard adopted in Nigeria for building and construction. The study revealed that 85% of all the samples tested have substandard strength and ductility due to non-compliance of carbon equivalence of tested samples to the given standards. They also confirmed that most of the locally produced rebars are non-weldable. This non-weldable property is contrary to the standard requirement for rebars [13].

In this study, the chemical composition and CEV of selected locally-rolled and imported reinforcement sample rods from Nigeria were examined. Their level of compliance was compared with the local, foreign and international standards. One-way analysis of variance (ANOVA) was used to ascertain whether there is a significant difference in chemical composition between and within the groups of selected brands in Nigeria.

## 2 Materials and methods

The selected reinforcement rods were procured from different open markets across Nigeria. This experimental research work analyses the chemical composition of 27 locally-rolled and three imported steel rods samples. Among the 27 locally-rolled samples, two were produced from imported billets (Brands 20B and 21), five (5) were Thermo-Mechanical Treated (TMT) (Brands 6B,11B,13B, 17B and 20A), and the other 20 rods named ordinary steel rods (Brands 1,2,3,4,5,6A,7,8,9,10,11A,12,13A,14,15,16,17A, 18,19 and 25) were produced without heat treatment. The three imported steels (Brands 22, 23 and 24) available in Nigeria markets were from Turkey, Brazil, and Ukraine. Some brands labelled with the same numerals but different alphabets (6A and 6B, 11A and 11B, 13A and 13B, and 17A and 17B) are from the same rolling mill company but are of different category types. The elemental composition data obtained from the selected rebars were analysed and compared with permissible deviations prescribed by five standards, namely SON [26] (Nigeria local standard), BSI [27], AISI 1018 [28], ASTM [29] (3 foreign standards) and ISO [30] (an international standard) standards. The tests were conducted according to the standards.

**Figure 1:**
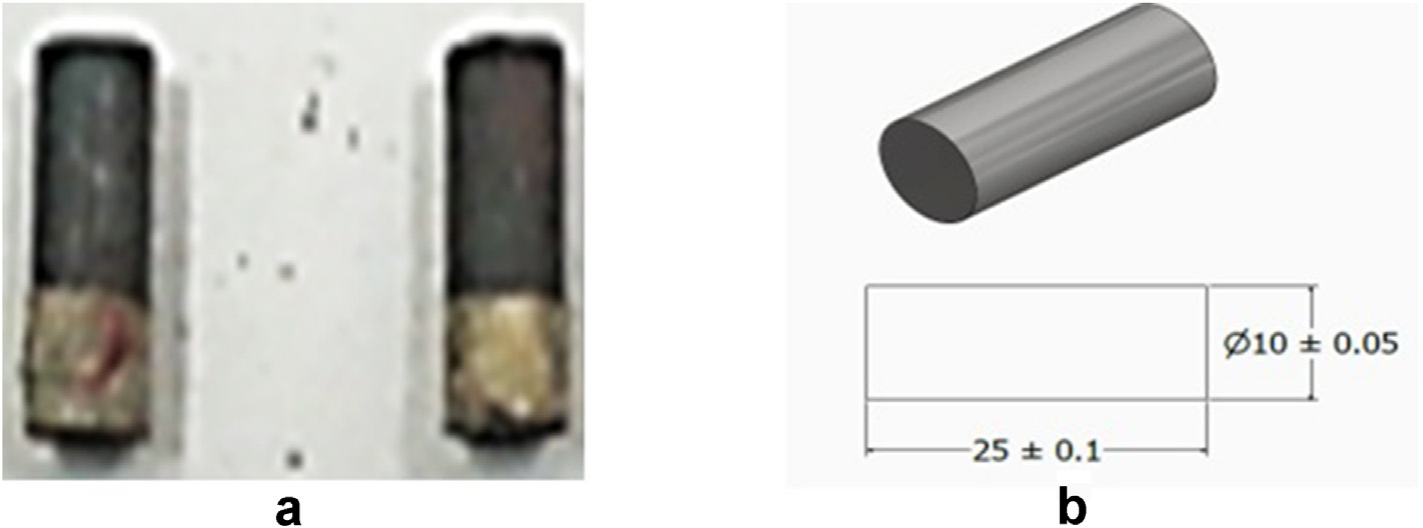
(a) Actual specimen for chemical composition and (b) Specimen diagram with measurement for chemical composition.

The chemical composition tests were carried out using an Optical Light Emission Spectrometer (Spectro-06000939; 120971 and Model no. Maxxlmf04) at Nigeria Foundries Limited in Lagos, Nigeria. The metal samples were first to cut into the required specimen size (Figure 1**).** These were polished using a metal surface polishing machine to obtain a smooth or flat surface without stains (oil, water, corrosion or holes). Each specimen was then placed on the spark stand of the spectrometer and clamped to allow the free flow of electrons through the sample. After sparking each specimen with inbuilt spectrometer electrodes, the chemical composition results were automatically obtained in the form of the average percentage of each element present.

The CEV_1_, was calculated according to Eq. (1), as stated by the selected standard documents SON, BSI, and ISO.

(1) $$CEV_{1}\;=\%C+\;\frac{\%Mn}{6}+\;\frac{\left(\%Cr+\%V+\%Mo\right)}{5}+\;\frac{\left(\%Cu+\%Ni\right)}{15}$$

Where C, Mn, Cr, V, Mo, Cu, and Ni are the mass fractions, expressed as percentages, of the respective chemical elements of the steel.

Conversely, CEV_2_ was calculated according to Eq. (2), as stated by the ASTM standard.

(2) $$CEV_{2}\;=\%C+\;\frac{\%Mn}{6}+\;\frac{\%Cu}{40}+\;\frac{\%Ni}{20}+\;\frac{\%Cr}{10}-\;\frac{\%Mo}{50}-\;\frac{\%V}{10}$$

For the data analysis, SPSS version 20 was used to perform a one-way analysis of variance (ANOVA) to determine whether there are any statistically significant differences in chemical composition between and within the groups at a 95% confidence level. The groups are Ordinary, TMT, locally-rolled from imported billets, and imported steel rods. In addition, Post Hoc Multiple comparisons tests were conducted to examine how the different steel groups are similar or dissimilar in terms of chemical composition.

The formulas (Eqs. (3), (4), (5), (6), (7), (8), (9), (10), and (11)) for the computation of the sum of squares, degrees of freedoms, and mean squares between and within groups are as follows [4]:

The sum of squares within groups is:

(3) $$SS_{w}\;=\sum_{J=1}^{k}\sum_{j=1}^{l}{\left(X-\;{\bar{X}}_{j}\right)}^{2}\;$$

The sum of the squares between the groups is:

(4) $$SS_{b}=\sum_{j=1}^{k}{\left(X_{j}-\;\bar{X}\right)}^{2}\;$$

The total sum of squares is:

(5) $$SS_{T}=\sum_{j=1}^{N}{\left(X_{j}-\;\bar{X}\right)}^{2}\;$$

where k = number of the group, j = number of all elements within the group, X = response of each element, $\bar{X}$ = mean of the samples, ${\bar{X}}_{j}$ = mean sample of each group and N = total number of observations.

Computation of various degrees of freedoms are as follows:

The degree of freedom within groups is:

(6) $$df_{w}=N-k$$

The degree of freedom between groups is:

(7) $$df_{b}=k-1$$

The total degree of freedom is:

(8) $$df_{T}=N-1=df_{b}+\;df_{w}$$

Computation of mean squares between and within groups are as follows:

The mean square within the groups is:

(9) $$MS_{w}\;=\;\frac{SS_{w}}{df_{w}}$$

The mean square between the groups is:

(10) $$MS_{b}\;=\;\frac{SS_{b}}{df_{b}}$$

Finally, the Fisher's ratio, or Mean-Square Ratio, is computed as:

(11) $$\text{F}\;=\;\frac{MS_{b}}{MS_{w}}$$

## 3 Results and discussions

**Figure 2:**
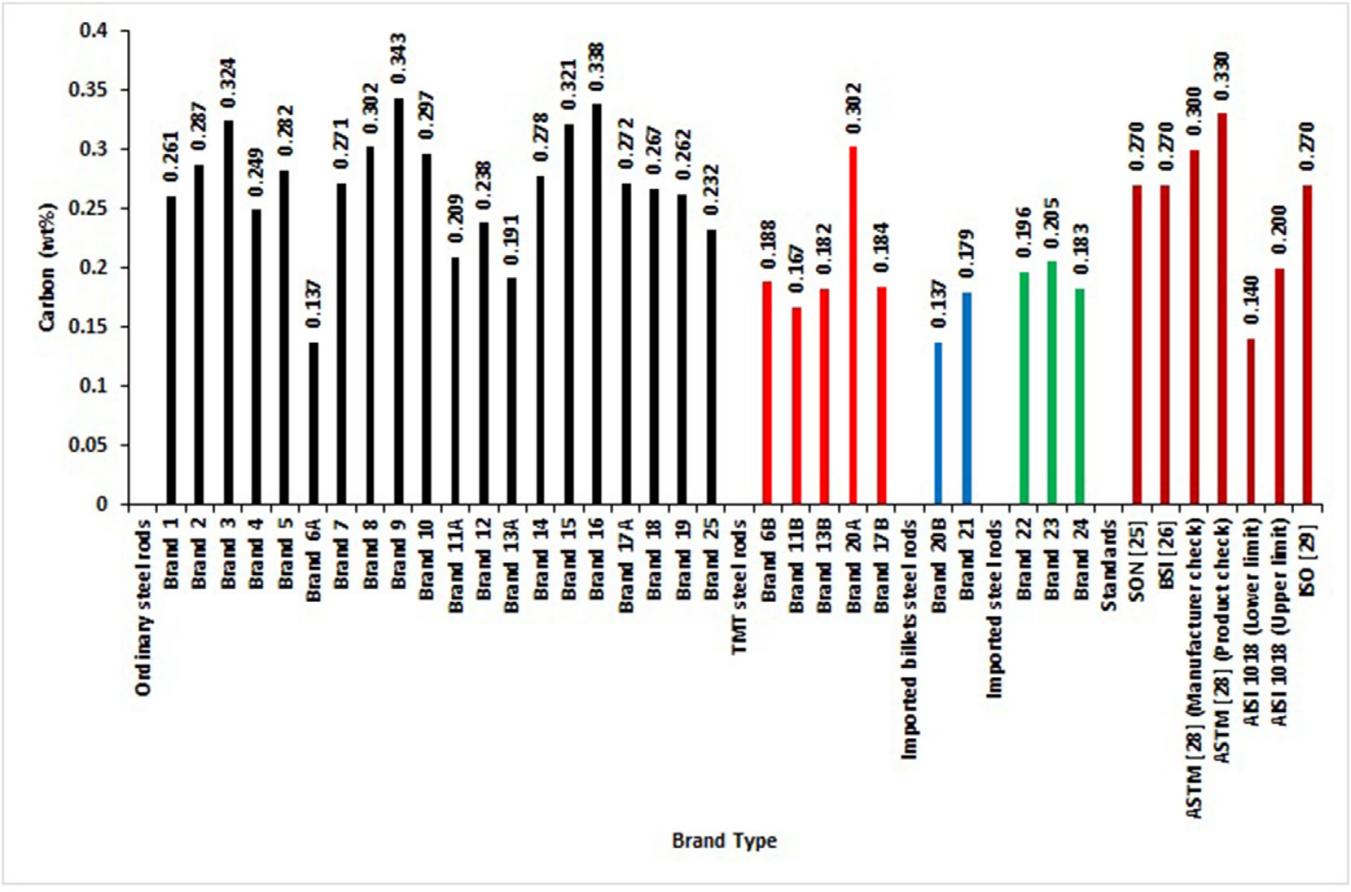
Carbon percentage by mass of different deformed steel rebar brands and maximum permissible deviation of local, foreign and international standards.

The carbon percentages by mass of the selected brands are presented in Figure 2. From Figure 2, it is observed that only 45.0% of ordinary, 80% of TMT, all locally-rolled with imported billets and imported reinforcement steel bars complied with SON and ISO standards specifications. The same trend of results were obtained for BSI standard if the condition of maximum permissible value of 0.27 for Carbon is satisfied (i.e. if CEV_1_ is reduced by 0.02). ASTM standard has two standards for checking Carbon content. One standard is for the manufacturer to inspect or test their sample during heat or Ladle analysis. In this standard, the Carbon content should not exceed 0.30%. The other standard is to be used by the purchaser of the steel bars during product check analysis. In this standard, the Carbon content should not exceed 0.33%. Therefore, for the manufacturer product check analysis, Brand 20A and Brands 3, 8, 9, 15, and 16 do not fall within the permissible region, but all imported and locally-rolled with imported billets brands fell within the allowable value for carbon inclusion in the rebar steel rods. For the other standard for purchaser product check analysis, all tested steel rods were within the stipulated limit except Brands 9, and 16 of the ordinary rolled steel rods. Considering AISI 1018 standard, 5% of ordinary, 50% of locally-rolled with imported billets, 66.7% of imported rods, and 80% of TMT steel bars fully complied with the acceptable ranges specified for Carbon content. It can be concluded that the imported deformed steel bars best complied with all the standards used in this work, followed by steel bars locally-rolled with imported billets. TMT and ordinary deformed steel bars were also followed in consecutive order to comply with carbon composition by mass in the selected standards. This conformance to standards requirements might be due to adherence to the quality control process. The criteria employed in each company during the production of these steel bars products can also affect the conformance to the standards. The trend of results obtained in this work was supported by many researchers [4, 6, 8, 11, 12].

**Figure 3:**
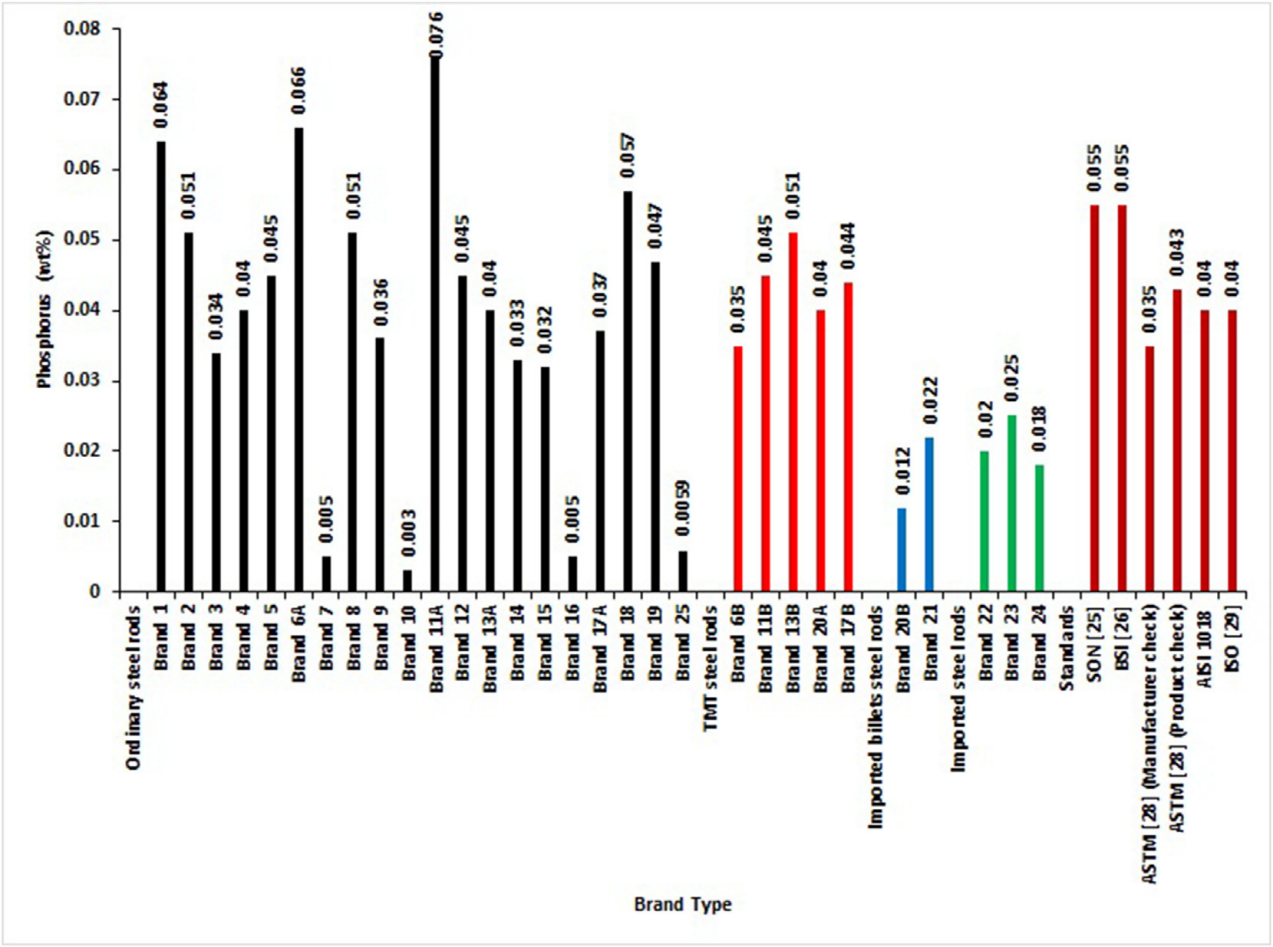
Phosphorus percentage by mass of different deformed steel rebar brands and maximum permissible deviation of local, foreign and international standards.

Figure 3 shows the percentage by mass of Phosphorus in different brands. For SON and BSI standards, all brands complied with permissible values of Phosphorus except Brands 1, 6A, 11A, and 18 of ordinary steel rods. Also, all imported and locally-rolled with imported billets complied with AISI 1018 and ISO standards, while 40% of TMT and 55% of ordinary reinforcement bars complied with these two standards. For ASTM standard, only Brand 6B of TMT, 35% of ordinary, all locally rolled with imported billets, and all imported steel rods were within the stipulated limits for manufacturers' product check analysis of Phosphorus content. Two of the TMT Brands (6B and 20A), 55% of ordinary, all locally rolled with imported billets, and all imported steel rods complied with specifications for product check condition. Odusote and Adeleke [11], and Adeleke et al. [12] also detected high content of Phosphorus using the same standards in their experimental research.

**Figure 4:**
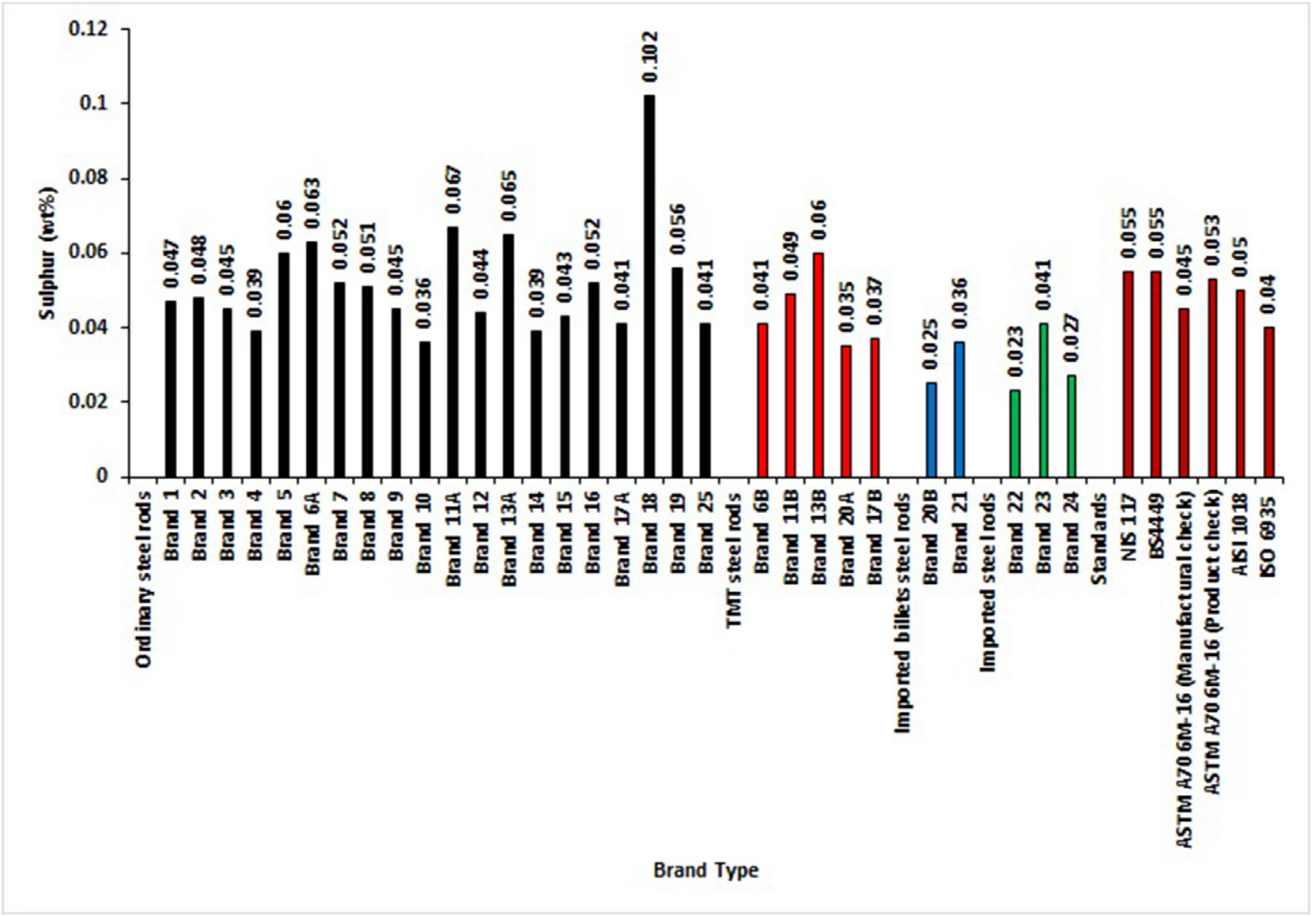
Sulphur percentage by mass of different deformed steel rebar brands and maximum permissible deviation of local, foreign and international standards.

Figure 4 shows that the elemental composition of Sulphur in all the tested brands complied with SON and BSI standards except Brands 5, 6A, 11A,13A, 18, 19 of ordinary steel rods and Brand 13B of TMT. With ASTM standard, all locally rolled with imported billet steel rods and all imported deformed steel rods complied with manufacturers' product check analysis specifications. Meanwhile, 55% of ordinary steel rods and Brands 11B and 13B did not comply with the standard. Likewise, all locally rolled with imported billet steel rods and all imported deformed steel rods were within the stipulated value for product check analysis, while 30% of ordinary steel rods and Brands 13B did not meet the specified limit. For AISI 1018 standard, only Brand 13B and 45% of ordinary steel rods did not comply with the maximum recommendation for Sulphur content. All other brands complied with the maximum permissible limit. With ISO standard, two brands of locally-rolled with imported billet steel rods fell within this international standard specification. Brand 23 of imported steel rods, 60% of TMT, and 85% of ordinary steel rods were not within the specified limit. The Sulphur percentage composition by mass showed that locally-rolled with imported billet steel rods complied with local, foreign and international standards. This was followed by all imported steel rebars and then TMTs and ordinary reinforcement rods. The deductions about Sulphur were confirmed in the studies of Adeleke et al. [12] and Adeleke and Odusote [14], who stated that Sulphur and Phosphorus contents are pretty higher in ISO standard than in BS4449, ASTM706 and NST-65-Mn standards. Alabi and Kayode [31] worked on the quality of steel rods from two cement production sites. They stated that the sulfur content of the steel rods samples was significantly lower compared to BS4449:2005, A707M-15, and Nst.65-Mn standards recommendations. These discrepancies may be due to improper quality control during production processes by the steel rods manufacturers. Sulphur and Phosphorus are the two chief lethal elements that must be controlled, if not, apart from hot-shortness and cold-shortness that may be resulted due to high content of Sulphur and Phosphorus respectively, the high presence of these duo elements can also reduce the ductility of bars thereby make the rods unsuitable for reinforcement of concrete [10, 14, 15].

**Figure 5:**
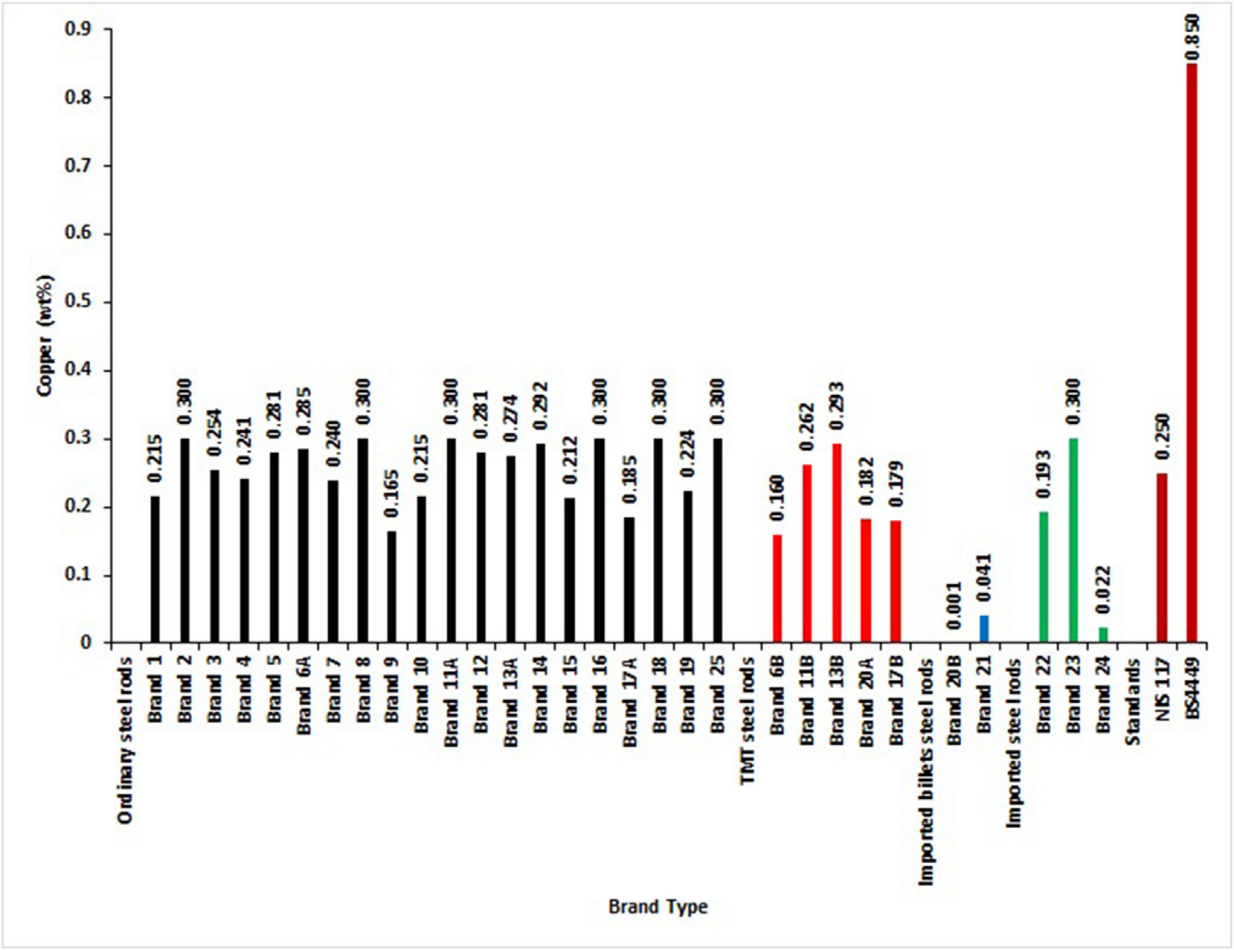
Copper percentage by mass of different deformed steel rebar brands and maximum permissible deviation of local, foreign and international standards.

For Copper composition by mass, Figure 5 shows that only SON and BSI standards have permissible values for Copper in the elemental composition of reinforcement steel rods. The samples test results showed that the two brands of locally-rolled with imported billets steel rods fully complied with SON standard. In contrast, Brand 23 of the imported steel rods, Brand 11B and 13B of TMTs, and 60% of locally-rolled with imported billets steel rods did not meet the permissible limits for Copper. For BSI standard, all brands fell within the specification limit. It can be concluded for Copper composition by mass that locally-rolled with imported billets steel rods met most of the requirements of the standard out of all the brands, followed by the imported steel rods, TMT, and ordinary steel rods.

The findings of Alabi and Onyeji [15] and Ponle et al. [16] were not different from these results of Copper composition for steel bars for the reinforcement of concrete available in Nigeria markets.

**Figure 6:**
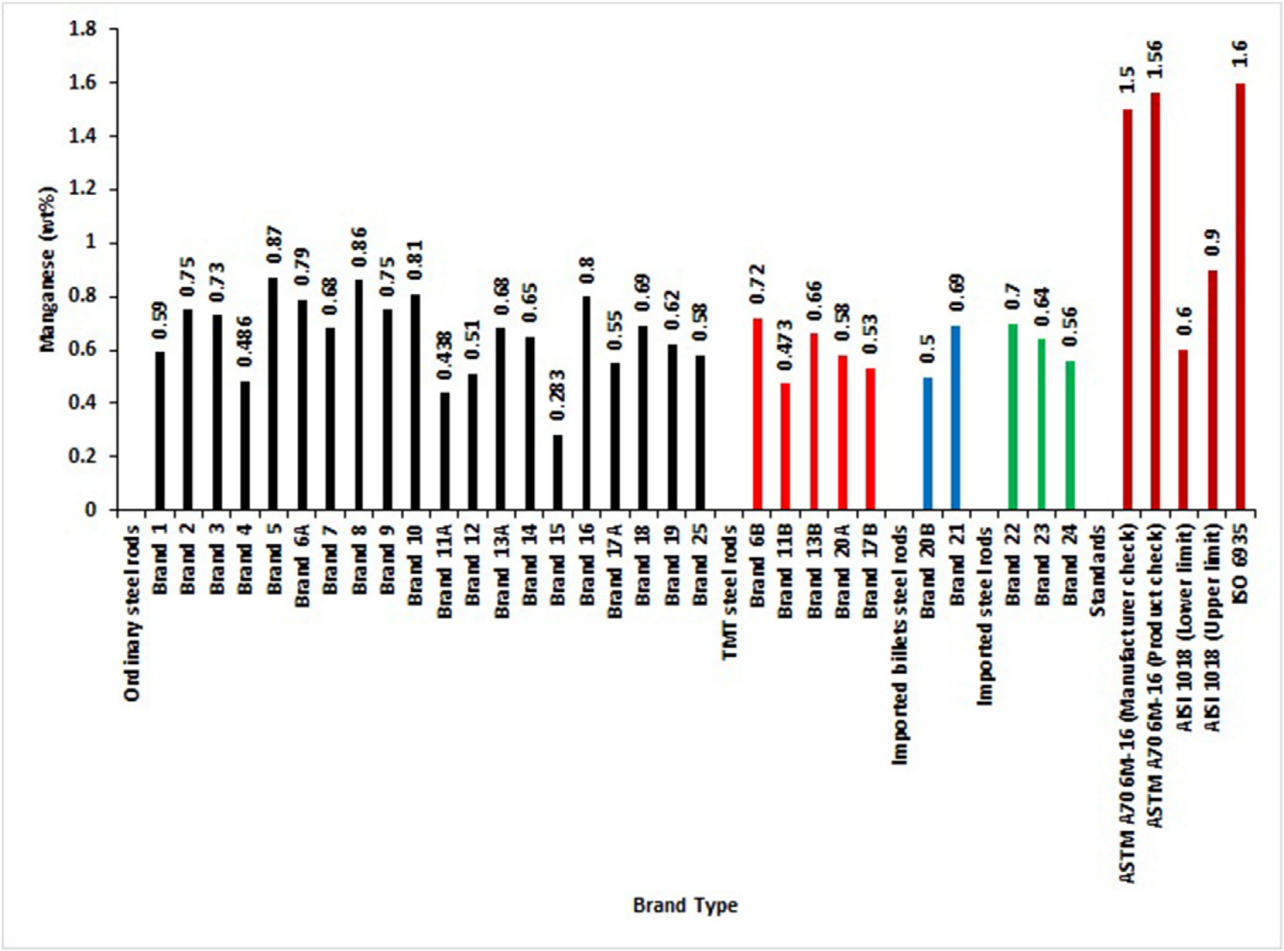
Magnesium percentage by mass of different deformed steel rebar brands and maximum permissible deviation of local, foreign and international standards.

The percentage composition by mass of Manganese (Figure 6) in chemical analysis of reinforcement rods only employed three out of five standards in this research. SON and BSI standards do not have specifications for Manganese for weldable steel rods. Nevertheless, Figure 6 revealed that all tested brands complied with ASTM (manufacturers and purchasers' products check analysis) and ISO standards specifications. The adherence to AISI 1018 standards specifications is given in descending order as 40.0% of TMT, 50.0% of locally-rolled with imported billets steel rods, 65.0% of ordinary and 66.7% of imported steels bars. In the characterization of a low carbon steel bar used to reinforce animal house buildings by Akinsola et al. [17], their results showed that Manganese agreed with the ASTM and ISO standards of the elemental compositions.

**Figure 7:**
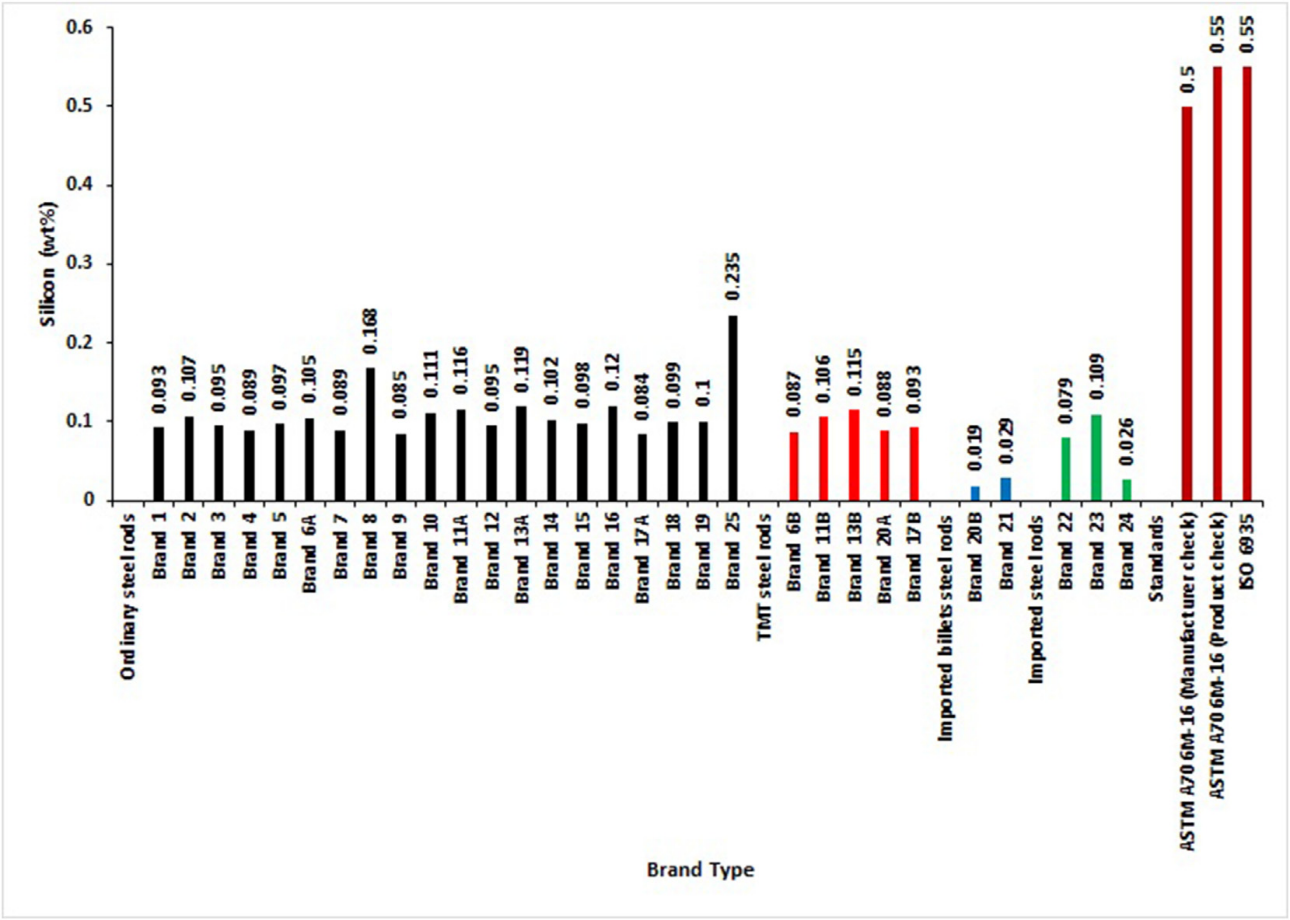
Silicon percentage by mass of different deformed steel rebar brands and maximum permissible deviation of local, foreign and international standards.

For evaluation of Silicon composition by mass in the selected brands, the result revealed in Figure 7 that not all the standards have specifications for weldable steel rebars. SON and BSI standards do not have Silicon specifications in their standard documents. The study also showed that all brands complied with ASTM (manufacturers and purchasers' product check analysis) and ISO standards acceptance limits. The same trend of results were obtained by Benneth and Julius [20] and Jibrin and Ejeh [21].

**Figure 8:**
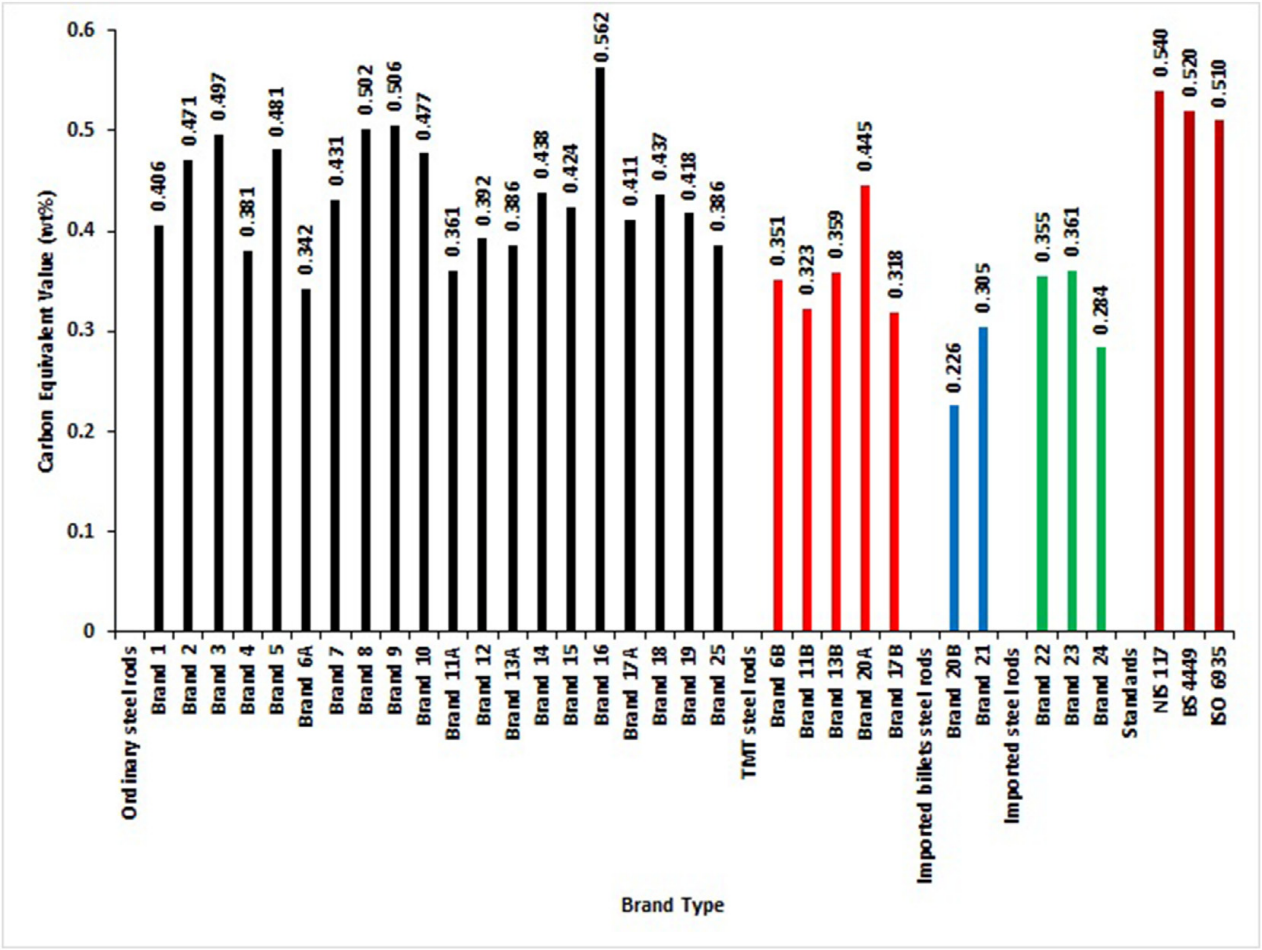
Carbon Equivalent Value (CEV_1_) of different deformed steel rebar brands and maximum permissible deviation of NIS 117, BS 4449 and ISO 6935 standards.

Figure 8 shows the plot of CEV_1_ data computed with Eq. (1). It depicted that only Brand 16 of ordinary steel rods did not meet the maximum limits specified for CEV_1_ in SON, BSI and ISO standards. The implication of non-compliance with these standards is that the brand cannot be considered weldable, the requirement for steel bars for reinforcement of concrete. Generally, from the computation of CEV_1_ in this research work, it was shown that most selected brands are weldable. This observation was contrary to the submission of Adeleke et al. [12] and Balogun et al. [13], who stated that most locally produced steel bars were non-weldable. These inconsistencies in the results may be due to the lack of proper control of the production process from the steel rods manufacturers.

**Figure 9:**
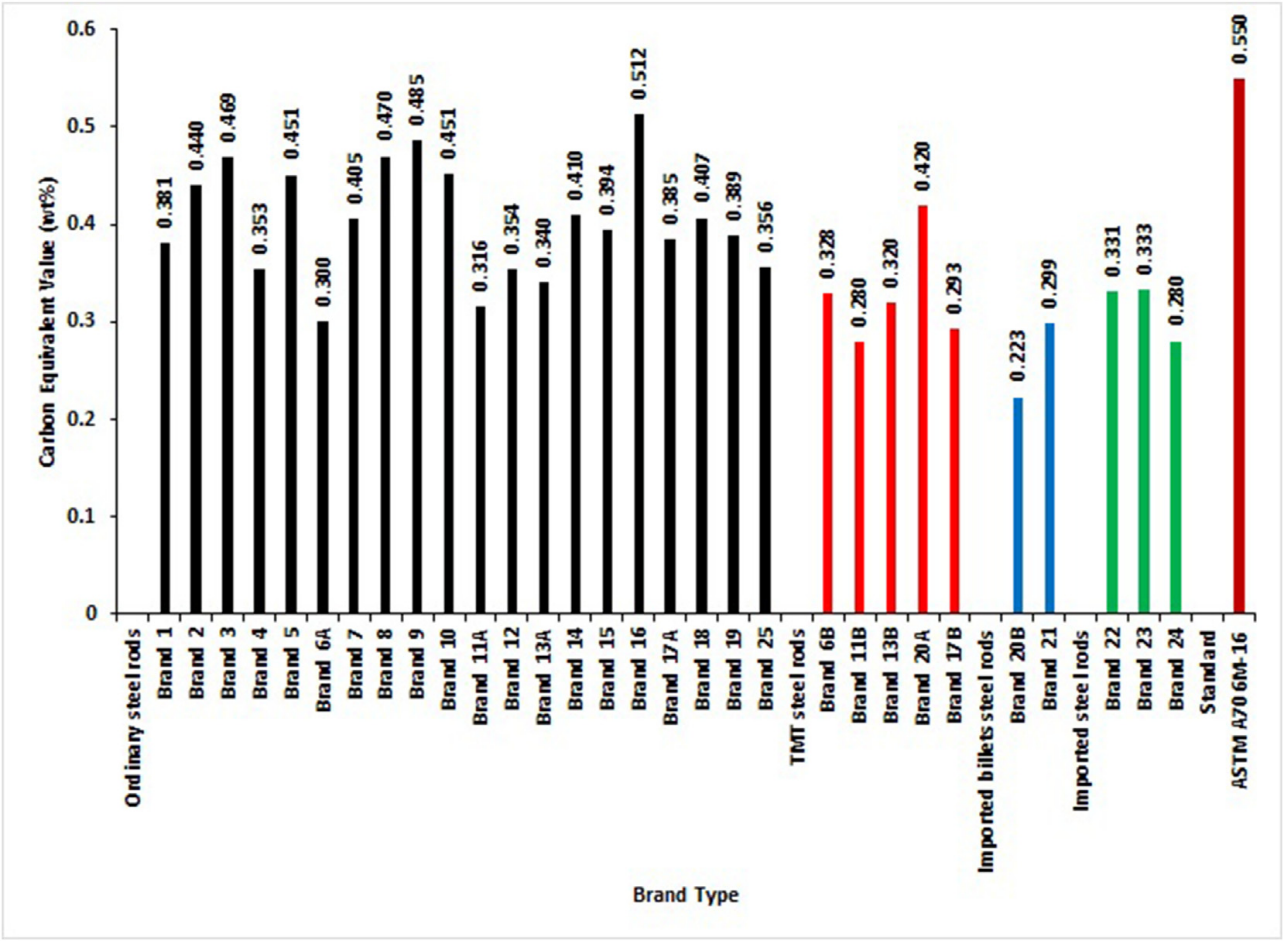
Carbon Equivalent Value (CEV_2_) of different deformed steel rebar brands and maximum permissible deviation of ASTM [29] standard.

Figure 9 shows the plot of CEV_2_ data obtained using Eq. (2). The results revealed that all of the selected brands met the conditions of the maximum permissible value of 0.55 CEV_2_ for the ASTM standard.

**Figure 10:**
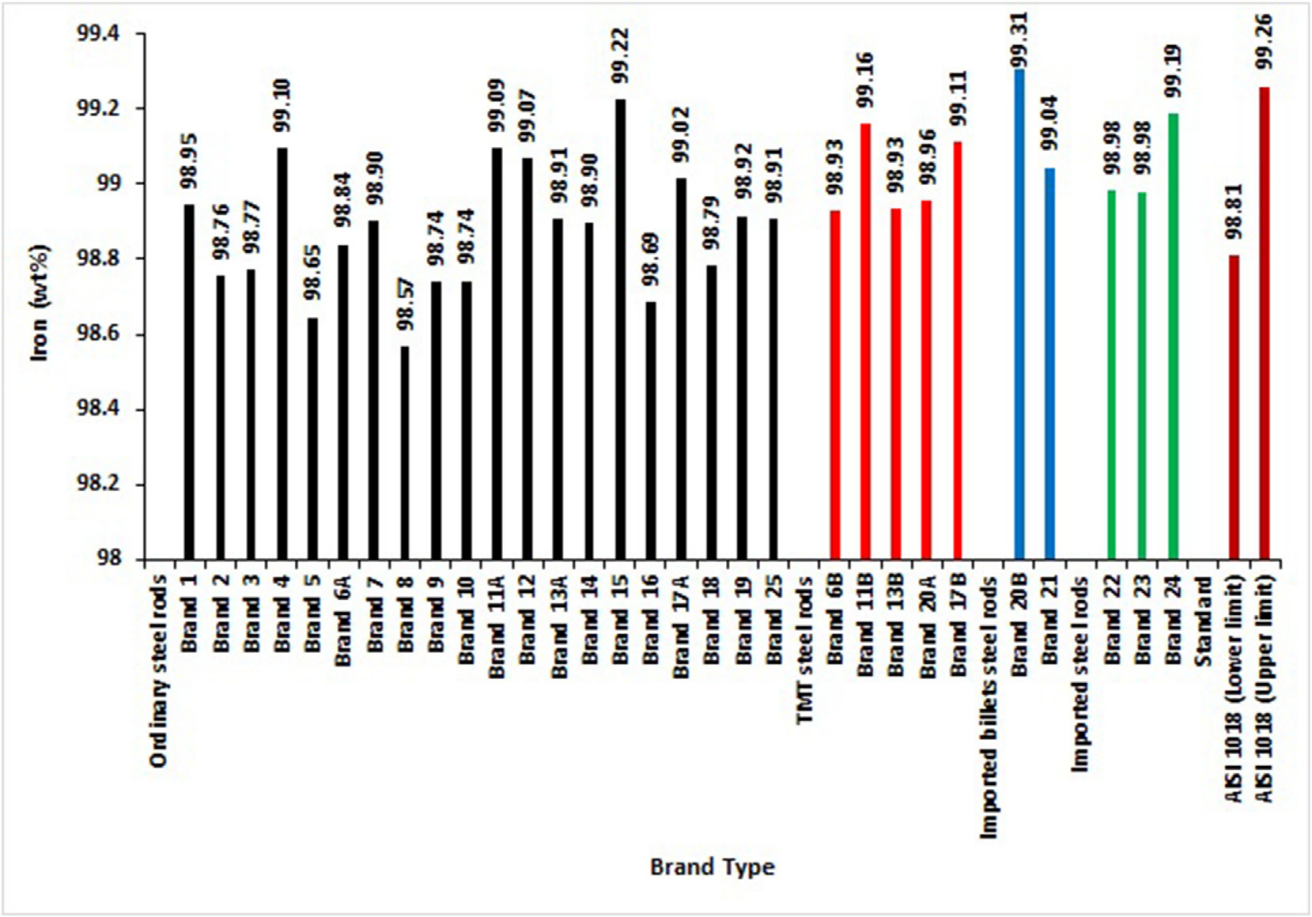
Iron percentage by mass of different deformed steel rebar brands and maximum permissible deviation of foreign standard (AISI 1018).

In evaluating the percentage of Iron content in the elemental composition of the selected brands, only AISI 1018 standard out of five chosen standards gave specification ranges for Iron. The results in Figure 10 revealed that Brand 21 of locally-rolled with imported billets steel rods, all TMTs and all imported steel rods complied with this standard. In contrast, 40% of ordinary steel reinforcement rods do not fall within the ranges specified for Iron. The results of Shuaib-Babata et al. [19], who evaluated the quality of reinforcement steel bars in some selected Nigerian markets, recorded that most locally-produced rods do not comply with AISI 1018 standard specification for Iron composition.

## 4 Chemical composition groups and brands analyses

The analysis of chemical composition between and within groups with ANOVA are performed with the data presented in Figures 2, 3, 4, 5, 6, 7, 8, 9, and 10. The One-Way Analysis of Variance (ANOVA) results in Table 1 reveal that at the degree of freedom of 3 (df = 3) and F = 19.27 (Eq.11), there is a significant difference (P < 0.05) in chemical composition and compliance level between the groups of selected concrete reinforcement steel rods rolled in Nigeria. Considering the mean value obtained for the different steel brand groups, the imported steel rods (mean = 101.4) have the highest mean value, followed by locally rolled from imported billets (101.2), TMT steel rods (101.0), and ordinary (100.6). These also show the sequence of performances in terms of the chemical composition of most of the selected reinforcement steel rods presented in Figures 2, 3, 4, 5, 6, 7, 8, 9, and 10.

**Table 1:** One Way ANOVA of chemical composition.

Group	Mean	Std. Deviation	F	Sig.
Ordinary	100.5974	.15379	19.274	.000∗
TMT	100.9745	.30295		
Locally rolled from imported billets	101.1758	.12412		
Imported steel rod	101.3716	.13798		
Total	101.2476	.26476		

Note: ∗Significant difference (P < 0.05). Sig. means significant level.

### 4.1 Post-Hoc multiple comparison test

Furthermore, a Scheffe Post-Hoc Multiple comparison test was performed on the chemical composition of different steel brand groups in Table 2. The results in the table reveal that at a 0.05 level of significance, the mean chemical composition of ordinary steel rods differs significantly from that of locally rolled from imported billets and imported steel rods (P < 0.05). Similarly, the mean chemical composition of TMT steel rods differs significantly (P < 0.05) from locally rolled from imported billets steel rods. Three homogenous subsets were generally derived through the Scheffe results (Table 3). The first classification has ordinary steel rods. The second contains locally rolled from imported billet steel rods and TMT, while the third contains imported steel rods and locally rolled from imported billets steel rods. These suggested that the chemical composition of ordinary steel rods are dissimilar (distinct) to that of the other steel groups. However, imported steel rods and locally rolled from imported billets have similar chemical compositions. Also, TMTs and locally rolled from imported billets have similar chemical compositions, thus being in the same homogenous subset.

**Table 2:** Scheffe multiple comparison on chemical composition of different steel brand groups.

(I) Brand	(J) Brand	Mean Difference (I-J)	Std. Error	Sig	95% Confidence Interval	
					Lower Bound	Upper Bound
Ordinary	TMT	.19580	.07787	.124	-.0368	.4284
	Locally rolled from imported billets	.77413∗	.11549	.000∗	.4291	1.1192
	Imported steel rod	.39703∗	.09642	.004∗	.1090	.6851
TMT	Ordinary	-.19580	.07787	.124	-.4284	.0368
	Locally rolled from imported billets	.57833∗	.13029	.002∗	.1891	.9676
	Imported steel rod	.20123	.11373	.390	-.1385	.5410
Locally rolled from imported billets	Ordinary	-.77413∗	.11549	.000∗	-1.1192	-.4291
	TMT	-.57833∗	.13029	.002∗	-.9676	-.1891
	Imported steel rod	-.37710	.14216	.096	-.8018	.0476
Imported steel rod	Ordinary	-.39703∗	. 09642	.004∗	-.6851	-.1090
	TMT	-.20123	.11373	.390	-.5410	.1385
	Locally rolled from imported billets	.37710	.14216	.096	-.0476	.8018

Note: ∗Significant difference (P < 0.05). Sig. means significant level.

**Table 3:** Homogenous subsets derived through the Scheffe results.

Scheffe Brand	Homogenous subsets		
	Subset for alpha = 0.05		
	1	2	3
Ordinary	100.5974		
TMT		100.9745	
Locally rolled from imported billets		101.1758	101.1758
Imported steel rod			101.3716
Sig.	1.000	.397	.421

Note: Sig. means significant level.

### 4.2 Mean composition of elements in different steel brands

Table 4 shows no significant difference (P > 0.05) in the composition of elements between ordinary, TMTs, locally-rolled from imported billets, and imported steel rods for Phosphorus, Manganese, and Molybdenum. Conversely, there is a significant difference in the elemental composition of Iron, Carbon, Sulphur, Copper, Silicon, Chromium, Vanadium, Nickel, CEV_1,_ and CEV_2_ in those groups. The significant difference implies a wide variation in the particular element in those groups. These also were shown in Figures 2, 3, 4, 5, 6, 7, 8, 9, and 10.

**Table 4:** One Way ANOVA for the mean of the groups’ elements of different steel brands.

Element	Group	Mean	Standard Deviation	F	Sig.
Fe	Ordinary	98.8753	.16816	3.480	.030∗
	TMT	99.0176	.10987		
	Locally rolled	99.1755	.18597		
	Imported steel rod	99.0493	.11836		
	Total	98.9364	.17698		
C	Ordinary	.2681	.05045	5.822	.003∗
	TMT	.2046	.05503		
	Locally rolled	.1580	.02970		
	Imported steel rod	.1947	.01106		
	Total	.2428	.05959		
P	Ordinary	.0386	.02090	1.813	.170
	TMT	.0430	.00596		
	Locally rolled	.0170	.00707		
	Imported steel rod	.0210	.00361		
	Total	.0362	.01884		
S	Ordinary	.0518	.01489	3.330	.035∗
	TMT	.0444	.01024		
	Locally rolled	.0305	.00778		
	Imported steel rod	.0303	.00945		
	Total	.0470	.01525		
Cu	Ordinary	.2582	.04314	11.099	.000∗
	TMT	.2152	.05853		
	Locally rolled	.0210	.02828		
	Imported steel rod	.1717	.14022		
	Total	.2266	.08375		
Mn	Ordinary	.6559	.15128	.352	.788
	TMT	.5926	.09894		
	Locally rolled	.5950	.13435		
	Imported steel rod	.6333	.07024		
	Total	.6390	.13421		
Si	Ordinary	.1104	.03461	5.188	.006∗
	TMT	.0978	.01224		
	Locally rolled	.0240	.00707		
	Imported steel rod	.0713	.04203		
	Total	.0986	.03854		
Cr	Ordinary	.1514	.05336	5.857	.003∗
	TMT	.1552	.04703		
	Locally rolled	.0230	.00707		
	Imported steel rod	.0700	.04359		
	Total	.1353	.06213		
Mo	Ordinary	.0141	.00740	1.553	.225
	TMT	.0142	.00968		
	Locally rolled	.0016	.00205		
	Imported steel rod	.0120	.01053		
	Total	.0130	.00817	3.266	.037∗
V	Ordinary	.0044	.00236		
	TMT	.0058	.00217		
	Locally rolled	.0015	.00071		
	Imported steel rod	.0017	.00058		
	Total	.0042	.00244		
Ni	Ordinary	.1046	.01871	10.914	.000∗
	TMT	.0978	.01224		
	Locally rolled	.0240	.00707		
	Imported steel rod	.0713	.04203		
	Total	.0948	.02905		
CEV_1_	Ordinary	.4355	.05631	9.290	.000∗
	TMT	.3593	.05113		
	Locally rolled	.2654	.05613		
	Imported steel rod	.3332	.04287		
	Total	.4012	.07443		
CEV_2_	Ordinary	.4035	.05795	6.871	.001∗
	TMT	.3283	.05478		
	Locally rolled	.2610	.05389		
	Imported steel rod	.3147	.02998		
	Total	.3726	.07055		

Note: ∗Significant difference (P < 0.05). Sig. means significant level.

## 5 Conclusions

The following conclusions were drawn from an experimental analysis of the chemical composition of the selected brands of steel bars available in Nigerian markets.

i. The study revealed discrepancies in the values of elements in the steel brands available in Nigerian markets due to non-compliance with standards specifications for steel bars for concrete reinforcement. This may lead to inadequacies in the expected properties of the reinforcement steel bars. Largely, the imported steel rebars and those reinforcement rods that are locally-rolled with imported billets meet standards specifications better than those local steel rods rolled from billets made of scraps obtained locally. The non-compliance of some required parameters in the elemental composition may be attributed to the manufacturers' poor quality control management of products.
ii. About 77.8% of locally-rolled steel rods are low-carbon steels because they contain 0.02–0.30% carbon by mass, while the remaining are medium-carbon steels since their carbon content by mass is between 0.30 and 0.90%. Meanwhile, local, foreign and international standards expected those brand types of steel rebars to be weldable low carbon steels from their specification requirements.
iii. Only some chemical elements of steel have specifications in the standards. Whereas, according to the chemistry of steel rods, those unmentioned elements may have an immense contribution to the service life of the rods even if they were only present in minute quantities.
iv. The ANOVA results show significant differences (P < 0.05) in chemical compositions among the groups of Ordinary, TMTs, locally rolled from imported billets and imported steel rods. Scheffe multiple comparisons of the chemical composition of different steel groups' results also revealed significant differences (P < 0.05) of the groups at a 95% confidence level.

## Recommendation

Standard Organisation of Nigeria (SON) in conjunction with Engineering, Building, and Architectural bodies, like the Council for the Regulation of Engineering in Nigeria (COREN), Nigerian Society of Engineers (NSE), Nigeria Institute of Builder (NIOB), Nigeria Institute of Quantity Surveyor (NIQS), Nigeria Institute of Architecture (NIA) and Architect Registration Council (ARCON) are to ensure absolute compliance with the standards specifications for the steel bars produced for the reinforcement of concrete by the manufacturers especially local standard: Nigerian Industrial Standard (NIS 117:2005) for the safety of resources such as finances, human lives, building structures, etc.

## Prime novelty statement

This manuscript addresses a comparative analysis of chemical composition and compliance level to established standards of concrete reinforcement steel rods rolled in Nigeria. To examine whether significant differences exist or not in the mean chemical composition for the different categories of selected steel rods, SPSS version 20 was used to perform an ANOVA test. Findings from this study show that at a 95% confidence level, there is a significant difference (P < 0.05) in chemical composition and compliance level between the different categories of selected steel rods. The imported steel rods recorded the highest mean (μ = 101.4) in terms of chemical composition and compliance, followed by locally rolled from imported billets (μ = 101.2), TMT steel rods (μ = 101.0), and ordinary steel rods (μ = 100.6). The study reveals discrepancies in the values of elements in the steel bands available in Nigerian markets due to non-compliance with the standards specifications.

Most importantly, Nigeria is a central hub for manufacturing and distributing reinforcement steels across sub-Saharan Africa. Therefore, it is not only of interest to design engineers and builders in Nigeria. Data findings from this study will interest nations that depend on Nigeria's steel products.

Researchers, engineers, builders, architects, and contractors can apply the data obtained for further usage. The values of the elemental data of rebars must comply with the standards; otherwise, it means a lot to concerned professionals.

## Declarations

### Author contribution statement

Richard O. Leramo: Conceived and designed the experiments; Performed the experiments; Contributed reagents, materials, analysis tools or data; Wrote the paper.

Lawson O. Adekoya: Conceived and designed the experiments; Performed the experiments; Wrote the paper.

Oluwaseun Kilanko, Stephen E. Eluwa, Phillip O. Babalola: Analyzed and interpreted the data.

Sunday O. Oyedepo, Samson O. Adeosun, Nicholas T. Leramo, Peter O. Aiyedun, Olayinka S. Ohunakin, Oluseyi O. Ajayi, Ojo S. I. Fayomi: Contributed reagents, materials, analysis tools or data.

### Funding statement

This research did not receive any specific grant from funding agencies in the public, commercial, or not-for-profit sectors.

### Data availability statement

Data included in article/supplementary material/referenced in article.

### Declaration of interests statement

The authors declare no conflict of interest.

### Additional information

No additional information is available for this paper.
